# High-Efficiency Bovine Sperm Sexing Used Magnetic-Activated Cell Sorting by Coupling scFv Antibodies Specific to Y-Chromosome-Bearing Sperm on Magnetic Microbeads

**DOI:** 10.3390/biology11050715

**Published:** 2022-05-06

**Authors:** Korawan Sringarm, Marninphan Thongkham, Supamit Mekchay, Chompunut Lumsangkul, Wannaluk Thaworn, Wiwat Pattanawong, Ekaphot Rangabpit, Pornchai Rachtanapun, Kittisak Jantanasakulwong, Anucha Sathanawongs, Surat Hongsibsong

**Affiliations:** 1 Department of Animal and Aquatic Sciences, Faculty of Agriculture, Chiang Mai University, Chiang Mai 50200, Thailand; korawan.s@cmu.ac.th (K.S.); marninphan_t@cmu.ac.th (M.T.); supamit.m@cmu.ac.th (S.M.); chompunut.lum@cmu.ac.th (C.L.); 2 Cluster of Research and Development of Pharmaceutical and Natural Products Innovation for Human or Animal, Chiang Mai University, Chiang Mai 50200, Thailand; 3 Cluster of Agro Bio-Circular-Green Industry, Faculty of Agro-Industry, Chiang Mai University, Chiang Mai 50100, Thailand; pornchai.r@cmu.ac.th (P.R.); kittisak.jan@cmu.ac.th (K.J.); 4 Faculty of Animal Science and Technology, Maejo University, Chiang Mai 50290, Thailand; wannaluk.333@gmail.com (W.T.); wpattanawong@gmail.com (W.P.); 5 Livestock Semen Production Center, Inthanon Royal Project, Department of Livestock Development, Ministry of Agriculture and Cooperatives, Chiang Mai 50360, Thailand; spcm_cmi@dld.go.th; 6 Faculty of Agro-Industry, Chiang Mai University, Chiang Mai 50200, Thailand; 7 Department of Veterinary Biosciences and Veterinary Public Health, Faculty of Veterinary Medicine, Chiang Mai University, Chiang Mai 50100, Thailand; anucha.sa@cmu.ac.th; 8 School of Health Sciences Research, Research Institute for Health Sciences, Chiang Mai University, Chiang Mai 50200, Thailand

**Keywords:** bull semen, sexing semen, magnetic-activated cell sorting, scFv antibody, semen quality

## Abstract

**Simple Summary:**

Female calves are favored for milk production and genetic advancement in the dairy industry, and sex selection by using sexed semen has been long considered. A potential alternative sperm sexing technique is magnetic-activated cell sorting combined with an immunological method that uses scFv antibodies against male-specific sites on Y-chromosome-bearing sperm; however, the technique should be evaluated for validity and accuracy. This study focuses on how well bovine sperm are separated by the use of magnetic microbeads coupled with scFv antibodies against Y-chromosome-bearing sperm (PY-microbeads). The results showed that sexed bovine sperm using PY-microbeads was a highly effective technique for distinguishing X- and Y-chromosome-bearing sperm. It had no negative impact on the quality of X-chromosome-bearing sperm. The technique produced 82.65% of X-chromosome sperm in the X-enriched fraction semen and 81.43% of Y-chromosome sperm in the Y-enriched fraction semen, which was utilized to generate target sexed bovine semen.

**Abstract:**

Sperm sexing technique is favored in the dairy industry. This research focuses on the efficiency of bovine sperm sexing using magnetic-activated cell sorting (MACS) by scFv antibody against Y-chromosome-bearing sperm (Y-scFv) coupled to magnetic microbeads and its effects on kinematic variables, sperm quality, and X/Y-sperm ratio. In this study, the optimal concentration of Y-scFv antibody coupling to the surface of magnetic microbeads was 2–4 mg/mL. PY-microbeads revealed significantly enriched Y-chromosome-bearing sperm (Y-sperm) in the eluted fraction (78.01–81.43%) and X-chromosome-bearing sperm (X-sperm) in the supernatant fraction (79.04–82.65%). The quality of frozen–thawed sexed sperm was analyzed by CASA and imaging flow cytometer, which showed that PY-microbeads did not have a negative effect on X-sperm motility, viability, or acrosome integrity. However, sexed Y-sperm had significantly decreased motility and viability. The X/Y-sperm ratio was determined using an imaging flow cytometer and real-time PCR. PY-microbeads produced sperm with up to 82.65% X-sperm in the X-enriched fraction and up to 81.43% Y-sperm in the Y-enriched fraction. Bovine sperm sexing by PY-microbeads showed high efficiency in separating Y-sperm from X-sperm and acceptable sperm quality. This initial technique is feasible for bovine sperm sexing, which increases the number of heifers in dairy herds while lowering production expenses.

## 1. Introduction

Livestock reproductive management aims to produce a high number of offspring that are good genetic breeders to expand production [1,2]. In the dairy industry, it is preferred to produce female calves for milk production and genetic improvement [3]. Sex preselection by using sexed semen has been considered. Bovine sperm sexing is a well-known technology. Various techniques have been applied to separate X-chromosome-bearing sperm (X-sperm) and Y-chromosome-bearing sperm (Y-sperm) based on the difference in their DNA content, which is 3.7–4.2% depending on the breed [1,2]. Originally, separating X and Y sperm was performed using flow cytometry combined with a cell sorter. Meanwhile, the so-called Beltsville sperm sexing technology has proceeded to the point of commercialization, with USDA licensee XY Inc. promoting it across the world [4]. This precise method is able to produce sex-sorted semen with an accuracy demonstrated by a female calve birth rate of 85–95% [2]. However, sperm sexing equipment is expensive to purchase and also to operate. Another major problem of cell sorting is that it increases the possibility of sperm deterioration due to the chemical and mechanical stress applied during the procedure, leading to low sperm quality and low fertility [5,6].

The immunological sexing approach is an alternate method of sexing sperm. Thongkham et al. (2021) [7] provided proof of principle for immunological sexing by using monoclonal antibodies (Mab) against male-specific sites on the plasma membrane of Y-sperm in order to identify between X- and Y-sperm, followed by a cytotoxic reaction that destroys Y-sperm, leading to a high X-sperm ratio in sexed semen. This sperm sexing technique has no damaging impact on acrosome integrity of the sperm or sexed sperm yields, and has a conception rate similar to that of conventional semen, with female calves born at a rate above 74%. The success of immunological sexing comes from the use of a highly specific antibody against an epitope on the plasma membrane of the sperm. A Mab generated against male-specific plasma membrane of Y-sperm produced by hybridoma cells [8] is a high-quality source of engineered recombinant antibodies, such as single-chain fragment variable antibodies (scFv antibodies). An scFv antibody structure is composed of variable regions of heavy (VH) and light (VL) chains of immunoglobulin, which are connected by flexible peptide linker such as (Gly_4_Ser)_3_ linker sequence. The protein engineering technique produces scFv antibodies by using recombinant bacteria. In addition, this technique has been utilized to improve the qualities of scFv antibodies, such as affinity and specificity [9,10]. Thaworn et al. (2020) [11] generated VH and VL genes and utilized them to generate a scFv gene (650 bp) that was expressed in *Escherichia coli* TG1 cells and produced a soluble scFv antibody specific for male-specific regions on the plasma membrane of Y-sperm. This soluble scFv antibody was advantageous for use in precisely sexing sperm to create a novel bovine semen sexing technique.

The immunological sexing technique was less expensive than flow cytometry cell sorting for sperm sexing and also produced a higher number of sperm per dose and better frozen-thaw sperm quality [7], but the accuracy (i.e., the female calf birth rate) was still lower than that of flow cytometry cell sorting [12]. Therefore, improving the accuracy of immunological sexing techniques remains a challenge.

The recently developed magnetic-activated cell sorting (MACS) technique uses specific antibody-conjugated magnetic microspheres to bind to a target antigen or protein so that different cell fractions can be separated by exposing the combined populations to a magnetic field [13]. A previous study showed that MACS effectively separated dead sperm and apoptotic sperm [14,15]. Said et al. (2006) [16] reported a 73.8% recovery rate after excluding spermatozoa with externalized phosphatidylserine utilizing paramagnetic annexin V-conjugated microbeads. Ybarra et al. (2016) [17] discovered that distinguishing membrane-damaged spermatozoa from viable sperm cells by biotin-labeled DNA aptamers with avidin-coated nanoparticles enhanced the selection procedure of sperm without impairing their potential to form embryos. Assumpção et al. (2021) [18] selected a process for high-quality sperm in bovine sperm utilizing MACS, that was quite effective in generating samples with a high percentage of viable cells, membrane integrity, and mitochondrial potential.

The use of MACS by coupling scFv antibodies against male-specific sites on magnetic beads may have the potential to serve as an alternative sperm sexing technique that improves upon the immunological sexing technique with more precision, less effect on sperm quality, and a lower cost of sexed sperm production. Therefore, the present study focuses on the efficiency of the sexing procedure in bovine sperm using MACS combined with scFv antibodies specific to the plasma membrane of Y-sperm coupled to the surface of the magnetic microbeads on sperm motility, kinematic variables, sperm quality, and the X/Y-sperm ratio.

## 2. Materials and Methods

### 2.1. Chemicals and Reagents

Magnetic microbeads coated with a poly (lactic acid) polymer film (PLA-M) with a surface carboxylic acid group (-COOH), a particle size of 30 µm, and a concentration of 10 mg/mL in suspension (micromod Partikeltechnologie, GmbH, Rostock, Germany) were used. The scFv antibody against male-specific sites on the plasma membrane of Y-sperm (Y-scFv antibody) was produced and prepared in our laboratory as described by Thaworn et al. (2020); this antibody presents a high binding capacity for Y-sperm and low cross-reactivity (4.25%) with X-sperm. *N*-hydroxysuccinimide (NHS), 1-ethyl-3-(3-dimethylaminopropyl)-carbodiimide hydrochloride (EDC), Dulbecco’s phosphate-buffered saline (DPBS), and 3,3′, 5,5′-tetramethylbenzidine (TMB) were purchased from Sigma Aldrich (USA). SYBR-14, propidium iodide (PI), Hoechst 33342, and lectin PNA from Arachis hypogaea (peanut) conjugated with Alexa Fluor 488 (PNA-Alexa 488) were purchased from Invitrogen (Eugene, OR, USA). Anti-HA tag peroxidase (Abcam, Cambridge, MA, USA), and commercially available sex-sorted X-sperm by cell sorter (GENEX, Shawano, WI, USA) were also used.

### 2.2. Estimation of the Amounts of Y-scFv Antibody Coupled to Magnetic Microbeads

The amount of Y-scFv antibody on the surface of the magnetic microbeads was evaluated. PLA-M microbeads (10 mg/mL) were added to each vial (50 µL), and then 400 µL of 0.1 mM EDC and 400 µL of 0.1 mM NHS were added and incubated for 4 h at 4 °C. Then, the carboxylic acid group on the surface of the magnetic microbeads was activated. After that, ten samples containing Y-scFv antibody solution were placed in each microbead vial to final concentrations of 0, 0.5, 1, 2, 4, 6, 8, 10, 12, 14, and 16 mg/mL, and each reaction volume was mixed thoroughly in a shaker incubator (SHKE480HP, Thermo Fisher Scientific, Kansas, MO, USA) at 4 °C overnight. After incubation, the microbeads coupled with scFv antibody (PY-microbeads) were trapped by a strong neodymium magnet and kept at 4 °C until use for the coupling capacity evaluation process.

In this situation, the coupling capacity of the Y-scFv antibody to the magnetic microbeads was determined by using an anti-HA tag. The magnetic microbeads coupled with scFv antibody at each concentration were washed five times with 0.2 mL of DPBS. Then, 20 µL of goat polyclonal anti-HA tag peroxidase (1:100,000) was added and incubated at room temperature for 1 h. The sample was subsequently washed five times with 200 mL PBS. After that, 100 µL of TMB solution was added as the substrate and kept for 15 min at room temperature. Then, adding 2 M sulfuric acid, the reaction was stopped, and the absorbance at 450 nm was determined using a SpectraMax M3 microplate reader (Molecular Devices, San Jose, CA, USA). The immunoreactivities were compared using optical density (OD) measurements.

### 2.3. Semen Sample Collection

Semen samples were taken from four tropical Holstein Friesian bulls aged 3–5 years and were kept separately at the Inthanon Royal Project Livestock Semen Production Center (Chiang Mai, Thailand). The Department of Livestock Development of the Thailand Ministry of Agriculture and Cooperatives has established legislation and regulations governing experimentation on live animals. Fresh sperm samples were obtained via an artificial vagina. The only semen samples that were used in the experiment had a sperm concentration of greater than 650 × 10^6^ cells/mL and total motility of greater than 80%.

### 2.4. Optimization of Y-scFv Antibody Coupling on Magnetic Microbeads for Sorting Sperm

The 10 mg/mL of PLA-M magnetic microbeads were activated with 0.1 mM EDC and 0.1 mM NHS. Then, six mixture samples were mixed with Y-scFv antibody solution in each microbead vial to final concentrations of 0, 0.5, 1, 2, 3, and 4 mg/mL, and each mixture was shaken thoroughly in a shaker incubator at 4 °C overnight. After incubation, the microbeads coupling scFv antibody (PY-microbeads) were trapped by a strong neodymium magnet. Then, the supernatant was removed, and the PY microbeads were collected. After that, the unbound carboxylic acid groups on the microbeads were enclosed by Tris (50 mmol/L, pH 7.4) for 15 min at room temperature, and the beads were then washed three times with DPBS and suspended in 100 μL of DPBS. The prepared PY-microbeads were kept at 4 ℃ until use in the evaluated sperm sexing procedure.

**Figure 1:**
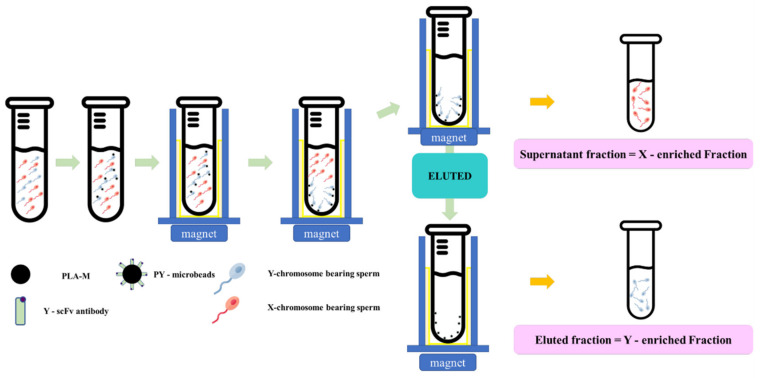
The schematic magnetic-activated cell sorting protocol for bovine sperm by using PY-microbeads.

Fresh semen was diluted in a Tris–citric acid-based extender to 4 × 10^6^ cells/mL. Then, each individual PY-microbead sample was added, and incubated for 20 min at 37 °C with gentle shaking and placed on a strong neodymium magnet for 5 min at room temperature. Then, unbound PY-microbeads, or the “supernatant fraction” were removed into new tubes. The sperm entrapped on PY-microbeads were separated from the microbeads using an eluting buffer (Tris–citric acid extender buffer containing 0.05 mM imidazole, pH 7.4), incubated for 20 min at 37 °C, and then placed on a strong neodymium magnet to trap the magnetic microbeads. The fractions eluted from the magnetic microbeads were transferred to new tubes and called the “eluted fraction”. The numbers of sperm in each “supernatant fraction” and “eluted fraction” were measured and used to indicate the PY microbead’s sperm binding capacity and sex sperm ratio. A schematic of the sperm sorting protocol using PY-microbeads is shown in Figure 1.

### 2.5. Production of Frozen Sexed Bovine Semen by PY-Microbeads

**Figure 2:**
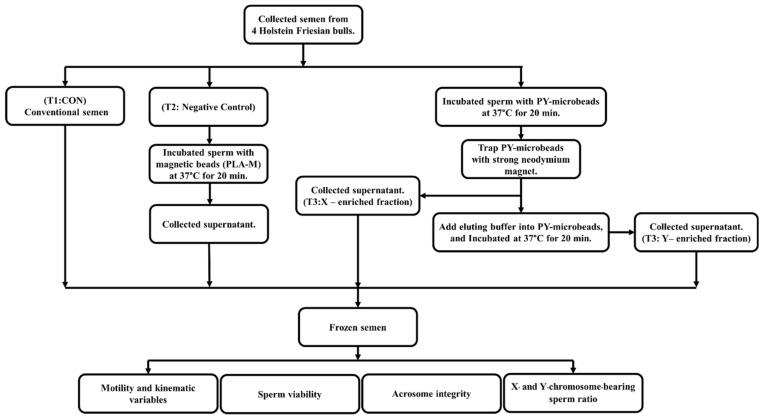
A diagram of the steps involved in magnetic-activated cell sorting for sexing bovine semen production and the evaluation of bovine semen in the present study.

Fresh semen samples from four tropical Holstein Friesian bulls were collected five times per bull using an artificial vagina. The first treatment was conventional semen treatment (T1; CON), in which fresh semen was diluted to a final concentration of 8 × 10^7^ cells/mL with a Tris–egg yolk-based extender (glycerol 8% *v*/*v*, egg yolk 20% *w*/*v*, D-Fructose 0.2% *w*/*v*, penicillin 1000 IU, and gentamycin 0.3 mg/mL). In the second treatment, fresh semen was incubated with PLA-M magnetic microbeads (unbound with scFv antibody) at 37 °C for 20 min and then diluted to a final concentration of 8 × 10^7^ cells/mL in Tris–egg yolk-based extender (T2; negative control; NC). The third treatment (T3) was sexed by using PY-microbeads (ratio 2 mg/mL Y-scFv: 10 mg/mL PLA-M beads), as follows: Fresh semen was diluted to a final concentration of 1 × 10^9^ cells/mL and incubated with PY-microbeads at 37 °C for 20 min. Then, the PY-microbeads, which captured Y-sperm, were trapped by a strong neodymium magnet. After that, unbound sperm were removed to new tubes (T3: X-enriched fraction). The sperm entrapped on the PY-microbeads were separated from the beads by incubation in the eluting buffer for 20 min at 37 °C. After that, the released sperm were transferred to new tubes (T3: Y-enriched fraction). Meanwhile, the X- and Y-enriched fractions were diluted to a final concentration of 8 × 10^7^ cells/mL with a Tris–egg yolk-based extender. In all treatments the sperm samples were frozen. A diagram of the protocol for bovine semen sexing and evaluation in the present study is shown in Figure 2.

### 2.6. Evaluation of Frozen–Thawed Sperm Motility and Kinematic Variables Using Computer-Aided Sperm Analysis (CASA)

The frozen semen samples from CON, NC, X-enriched, and Y-enriched fractions were thawed for 30 s in a water bath at 37 °C. AndroVision software (Minitube of America—MOFA^®^, Verona, WI, USA) linked to a Zeiss AxioScope with a heated stage at 37 °C (Carl Zeiss MicroImaging GmbH, Göttingen, Germany) was used to assess motility and kinematic characteristics. Aliquots of sperm (5.0 µL) were deposited on prewarmed slides, covered with a coverslip, and then subjected to rapid CASA analysis. The following parameters were measured and statistically analyzed in this study: total sperm motility (TM, %), progressive sperm motility (PM, %), distance average path (DAP, µm), distance curved line (DCL, µm), distance straight line (DSL, µm), velocity average path (VAP, µm/s), velocity curved line (VCL, µm/s), velocity straight line (VSL, µm/s), beat cross frequency (BCF, Hz), STR straightness VCL/VAP (STR, %), linearity VSL/VCL (LIN, %), and wobble VAP/VCL (WOB) [19].

### 2.7. Evaluation of Frozen–Thawed Sperm Viability Using Imaging Flow Cytometry

The viability of sperm in frozen semen straws from each treatment was assessed using two fluorescent probes: SYBR-14 for living sperm and PI for dead sperm, as described by Thongkham et al. (2021) [7]. After thawing, the sperm samples were centrifuged at a speed of 269× *g* for 8 min. Then, 300 mL of PBS containing 2 × 10^6^ sperm was incubated for 10 min in the dark with 1.2 µL of diluted SYBR-14 and 3 µL of PI (2.4 mM) before imaging flow cytometry analysis (FlowSight, Seattle, WA, USA). SYBR-14 and PI were stimulated using a 60 mW 488-nm laser. IDEAS 6.2 was used to examine the data (Amnis, Seattle, WA, USA).

### 2.8. Evaluation of Frozen–Thawed Sperm Acrosome Integrity Using Imaging Flow Cytometry

The acrosome integrity status of sperm from each treatment was determined following Thongkham et al. (2021) [7]. After thawing, the sperm samples were centrifuged at 269× *g* for 8 min. Then, PNA-Alexa 488 (100 µg/mL) of 0.5 µL and PI (1 mg/mL) of 0.5 µL were added to each sample (final concentration of sperm 1.5 × 10^6^ sperm/mL), and the mixture was incubated for 10 min at 37 °C. After that, the samples were centrifuged at 1075× *g* for 5 min, the supernatant was removed, and the pellets were resuspended in 50 µL of PBS before imaging flow cytometry analysis (FlowSight). The IDEAS version 6.2 (Amnis, Seattle, WA, USA) program was utilized for data analysis. A 60-mW 488-nm laser was used to stimulate PNA-Alexa 488 and PI.

### 2.9. Discrimination of X/Y-Sperm by Imaging Flow Cytometry

The X/Y sperm ratio in each treatment was determined following Thongkham et al. (2021) [7]. After thawing the semen sample, semen samples were diluted to 1 × 10^6^ sperm/mL with PBS mixed with 1.2 μL of Hoechst 33,342 (50 μg/mL). Then, the sample was incubated for 10 min at 37 °C in the dark and the results were analyzed using image flow cytometry (FlowSight). A 15-mW 405-nm laser was used to stimulate the Hoechst 33,342 stain. Histograms were produced to evaluate the fluorescence of Hoechst 33,342.

### 2.10. Evaluation of Sexed Sperm by SYBR^®^ Green RT–PCR

A pair of specific primers were constructed for the bovine Y and X chromosomal partial sequences using the NCBI website and the SYBR^®^ Green Real-Time PCR settings. As a positive control, the internal housekeeping gene GAPDH was used. The pair of Y-specific primers was constructed to target a conserved region of the SRY gene connected to the bovine chromosome. For the bovine proteolipid protein gene, primers specific to the X chromosome were created (PLP). The forward and reverse primer sequences, Tm, and amplicon length are listed in Table 1 [20].

#### 2.10.1. DNA Sample Preparation

DNA was extracted from each semen sample by using the protocol of Chandler et al. (2002) [21] with some modifications. Sperm from 5 straws from each treatment were thawed and washed twice with D-PBS (composition). Proteinase K (Qiagen Inc., Valencia, CA, USA), 1 M dithiothreitol (DTT) in 0.01 M sodium acetate, and 5% Chelex (Sigma Aldrich (USA) were added to the samples. The samples were gently mixed and incubated at 37 ℃. After incubation, the samples were extracted with DNA using the QIAamp^®^ DNA mini kit (Qiagen, Valencia, CA, USA). The procedures for DNA extraction were carried out according to the manufacturer’s instructions. Quantification of the samples was performed using a NanoDropTM 2000 (Thermo Scientific, Wilmington, DE, USA) at an absorbance ratio of 260–280 nm. The sequences of the forward and reverse primers and amplicon length are given in Table 1. GAPDH was used as an internal housekeeping gene.

#### 2.10.2. Quantitative SYBR Green Real-Time PCR (qPCR) Analyses

Real-time PCR was performed on a CFX Connect™ Real-Time PCR System by using iTaq Universal SYBR Green Supermix (2X) (Bio–Rad Laboratories, CA). DNA (25 ng) was performed in triplicate along with a control for each test. Amplification was performed by an optimized protocol (5 min at 95 °C, 40 repeated cycles of two steps at 95 °C for 15 s, 55 °C X gene/56 °C Y gene for 15 s and 72 °C for 30 s).

### 2.11. Statistical Analysis

Motility and kinematic variables, acrosome integrity, percentages of X- and Y-sperm, and evaluation of sexed sperm by SYBR^®^ Green RT–PCR were analyzed by one-way ANOVA using the statistical software program SPSS version 20.0 (SPSS Inc., Chicago, IL, USA). Duncan’s new multiple range test was used to compare values between individual groups [7,22]. Data are presented as the mean ± standard deviation. A paired *t*-test was used to compare X- and Y-chromosome-bearing sperm on a histogram as a double Gaussian distribution.

## 3. Results

### 3.1. Efficiency of Y-scFv Antibody Coupling to Magnetic Microbeads

**Figure 3:**
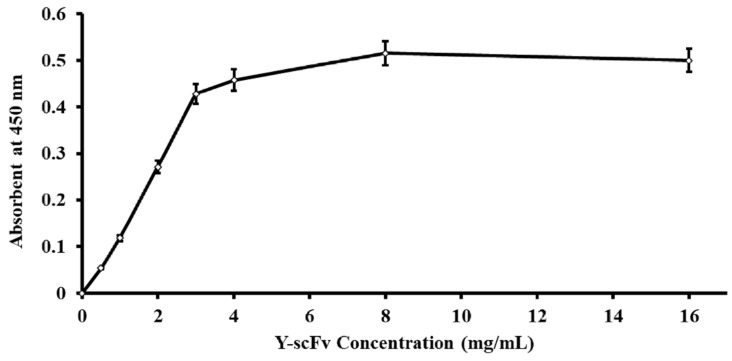
Correlative amount of Y-scFv antibody coupled to the surface of PLA-M magnetic beads.

The amount of Y-scFv antibody coupled to the surface of the magnetic microbeads was determined by using goat polyclonal anti-HA tag peroxidase that reacted directly to the HA position on the scFv antibody. The concentration of Y-scFv antibody bound to 10 mg magnetic microbeads was increased stepwise until saturation at 3–16 mg/mL. The effectiveness of Y-scFv antibody coupling on the surface of the magnetic microbeads in the range of 2–4 mg/mL was tested for sperm sexing, as shown in Figure 3. According to this result, the Y-scFv antibody was fully bound to the surface of the PLA-M magnetic microbeads by using the EDC–NHS reaction.

### 3.2. Optimized Conditions for Sexed Sperm by PY-Magnetic Beads

**Figure 4:**
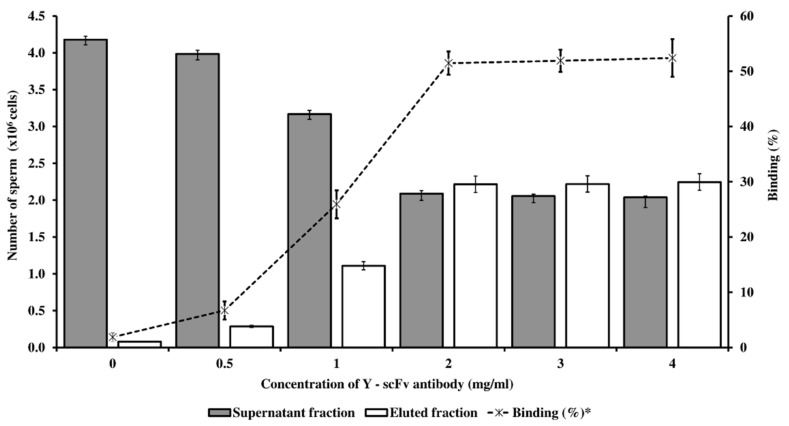
The concentration of Y-scFv antibody coupled to PLA-M microbeads bound with conventional bovine sperm with an X/Y-sperm ratio of 1:1 (expressed in the bar graph) and percentage of binding capacity (expressed in the linear graph) in fresh semen. * % Binding = (Eluted fraction sperm/total sperm) × 100.

The coupling of Y-scFv antibody on the surface of magnetic microbeads was performed via EDC–NHS. The subsequent process consisted of the specific targeting and removal of Y-sperm by the Y-scFv antibody coupled to the magnetic microbeads. Selected concentrations of Y-scFv antibody were coupled to the surface of the magnetic microbeads, and as shown in Figure 4, increasing the concentration of Y-scFv antibody significantly increased the percentage of sperm binding, compared with that bound by uncoupled PLA-M microbeads (0 mg/mL Y–scFv). Furthermore, magnetic microbeads coupled with 2–4 mg/mL Y-scFv demonstrated a high sperm binding efficiency of up to 51.5–52.4% of total sperm samples with an X/Y-sperm ratio of 1:1 (Figure 4).

The X/Y-sperm ratio in each fraction is shown in Table 2. According to these results, the high efficiency of PY-microbeads binding to Y-sperm was demonstrated when a high concentration of Y-scFv antibody was coupled to the PY-microbeads, leading to a high proportion of Y-sperm in the eluted fraction. In particular, a Y-scFv antibody concentration of 2–4 mg/mL produced significant enrichment of Y-sperm in the eluted fraction (78.01–81.43%) and of X-sperm in the supernatant fraction (79.04–82.65%). The optimal Y-scFv antibody concentration on PY-microbeads appropriate for bovine sperm sexing was indicated as 2 mg/mL.

### 3.3. Frozen–Thaw Sperm Motility and Kinematic Variables Evaluated by CASA

The percentage motility and kinematic variables of frozen–thawed sperm in CON, NC, the X-enriched fraction, and the Y-enriched fraction are shown in Table 3. After sexing by PY-microbeads, the motility and kinematic variables of the X-enriched fraction did not differ from those of CON and NC (*p* > 0.05). However, the motility and kinematic variables of the Y-enriched fraction were significantly lower (*p* < 0.05) than those of CON, NC, and the X-enriched fraction.

### 3.4. Viability of Frozen–Thawed Sexed Semen

**Figure 5:**
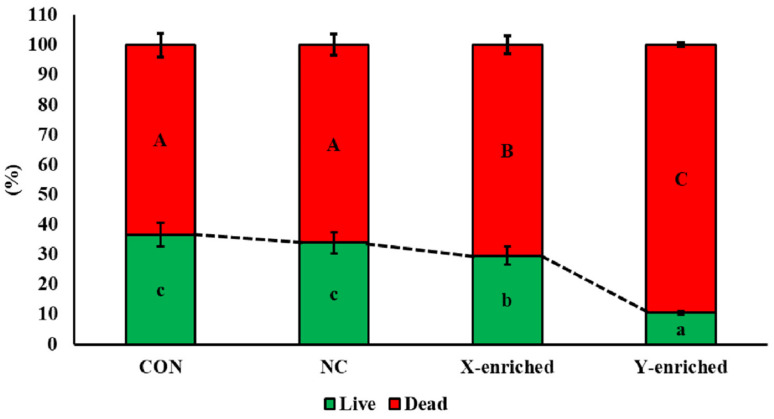
The percent viability of frozen–thawed sexed semen by PY-microbeads. Con: conventional frozen–thawed semen; NC: conventional semen incubated with nonactive PLA-M microbeads; X-enriched: sperm unbound by PY-microbeads; Y-enriched: sperm entrapped by PY-microbeads; A, B, C = comparison of dead sperm in each group; a, b, c = comparison of live sperm in each group.

The viability of frozen–thawed sperm in CON, NC, the X-enriched fraction, and the Y-enriched fraction, is shown in Figure 5. The percentages of live sperm were significantly higher in CON (36.7%) and NC (33.8%) than in the X-enriched fraction (29.5%) and Y-enriched fraction (10.5%) (*p* < 0.05). In addition, the percentage of live sperm in the X-enriched fraction was significantly higher than in the Y-enriched fraction (*p* < 0.05).

### 3.5. Acrosome Integrity of Frozen–Thawed Sexed Semen

The percentage acrosome integrity of frozen–thawed sperm in CON, NC, X-enriched fraction and Y-enriched fraction is shown in Table 4. Live acrosome-intact sperm (LI) and dead acrosome-intact sperm (DI) in X-enriched sperm did not differ between CON and NC (*p* < 0.05). The proportion of live acrosome-reacted sperm (LR) in X-enriched fraction was significantly lower than in CON and NC (*p* < 0.05) but not significantly different from in the Y-enriched fraction (*p* > 0.05). Moreover, the proportions of live acrosome-intact sperm and live acrosome-reacted sperm were significantly lower in the Y-enriched group than in CON and NC (*p* < 0.05). The level of dead acrosome-intact sperm (DR) in the Y-enriched fraction was significantly higher than in CON, NC, and the X-enriched fraction. The proportions of dead acrosome-reacted sperm showed no differences among CON, NC, X-enriched fraction, and Y-enriched fraction.

### 3.6. X/Y-Sperm Ratio in Frozen-Thaw Semen Evaluated by Imaging Flow Cytometry

**Figure 6:**
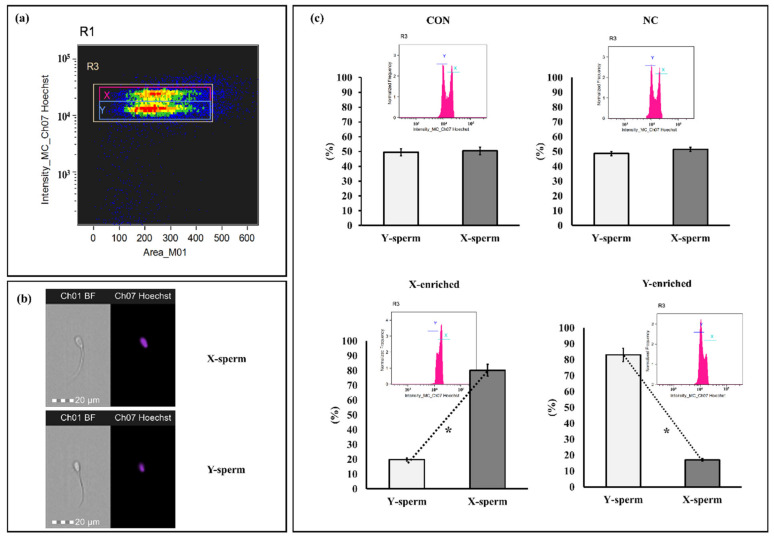
Discrimination between X- and Y-sperm after sexing by PY magnetic beads. (**a**) Regions are utilized to analyze sperm bearing the X and Y chromosomes. Separate populations of sperm bearing X and Y chromosomes were isolated, and the sex ratio in each sample was evaluated. (**b**) Patterns of sperm were observed with Hoechst 33,342. (**c**) Frequency histogram and percentage of sperm after staining with Hoechst 33,342. Con: conventional frozen–thawed semen; NC: conventional semen incubated with inactive magnetic PLA-M; X-enriched: fraction of sperm not bound by PY-magnetic beads; Y-enriched: fraction of sperm entrapped by PY-magnetic beads. * Paired *t*-tests were used to compare X- and Y-sperm from each group. *p*-value < 0.05 is statistically significant.

The sperm sex ratios calculated for frozen–thawed CON, NC, X-enriched fraction, and Y-enriched fraction are shown in Figure 6. The percentages of X- and Y-sperm did not differ between the CON and NC groups (*p* > 0.05). However, the percentage of X-sperm in X-enriched fraction (80.24 ± 3.85) was significantly higher than that of Y-sperm (19.76 ± 3.81) (*p* < 0.05). Conversely, the percentage of Y-sperm in the Y-enriched fraction (81.45 ± 4.95) was significantly higher than that of X-sperm (18.55 ± 4.91) (*p* < 0.05).

### 3.7. X/Y-Sperm Ratio in Frozen-Thaw Semen Evaluated by Real-Time PCR

**Figure 7:**
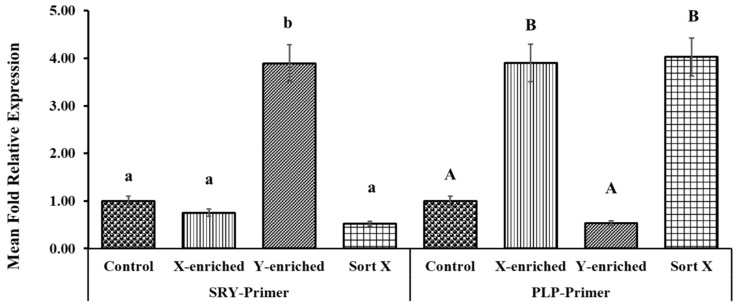
Expression levels of PLP and SRY genes in X- and Y-enriched semen samples were measured by RT–PCR to validate the sexing technique for bull semen. Con: conventional frozen–thawed semen; X-enriched: fraction of sperm not bound by PY-magnetic beads; Y-enriched: fraction of sperm entrapped by PY-magnetic beads; Sort X: commercial cell-sorter-sexed semen; A, B = comparison of PLP amplicons from each group; a, b = comparison of SRY amplicons from each group.

The fold relative expression data for X- and Y-sperm content obtained using real-time PCR analysis are depicted in Figure 7. CON was taken as the baseline. The mean fold relative expression of Y-sperm in the Y-enriched fraction sexed by the PY-microbeads method was significantly higher (*p* < 0.05) for the Y primer (SRY) and the average 2 delta Ct value for the Y primers was significantly higher (4-fold) for the Y primers compared with the control. The mean fold relative expression of X-sperm in the Y-enriched group was lower (0.52-fold) (*p* < 0.05) for the Y primer and the results were comparable to those in the control. The mean fold relative expression of X-sperm in X-enriched sperm sexed by PY-microbeads was higher (*p* < 0.05) for the X primer (PLP), as was the average 2 delta Ct value for X primers (3–4-fold) compared with CON. In addition, the mean fold relative expression of X-sperm in X-enriched semen sexed by PY-microbeads did not differ from that of commercial cell sorter-sexed (SORT X) semen (4-fold, *p* > 0.05). However, the mean fold relative expression of Y-sperm in X-enriched fraction was significantly lower (*p* < 0.05), and the average 2 delta Ct value for the X primers was significantly lower than the control.

## 4. Discussion

Sexed semen has successfully been used in bovines and has revolutionized the dairy industry [23]. The most common use of sexed semen is for the sex preselection of females to achieve an adequate number of replacement heifers is [15]. Utilizing sexed sperm is a successful method of obtaining offspring of a specific gender [24]. Many previous studies have demonstrated that MACS is highly effective in separating sperm based on plasma membrane integrity [16,17]. The present work was the first MACS to use scFv antibodies specific to plasma membrane epitopes on Y-sperm coupled to magnetic microbeads to separate X- and Y-sperm in bovine semen. The advantage of producing scFv antibodies against Y-sperm is that it creates smaller protein molecules than monoclonal antibodies with a high affinity to capture Y-sperm [11]. Therefore, MACS, by using scFv antibodies specific to Y-sperm coupled to the surface of magnetic microbeads, is an interesting approach that might be utilized to develop bovine semen sexing technologies. The coupling of antibodies to the surface of PLA-magnetic microbeads via 1-(3-dimethylaminopropyl)-3-ethylcarbodiimide hydrochloride-N hydroxysuccinimide (EDC–NHS) activation involves a two-step procedure. First, alkane carboxylic acids containing thiols are bound to the particle surface to generate a carboxylic acid-terminated monolayer. Second, the terminal carboxylic acids are coupled with peptides or antibodies by EDC–NHS coupling to generate amide linkages [25]. Both the sperm binding efficiency of this method and its ability to produce high-quality sperm have been verified.

The approaches for estimating the amount of Y-scFv antibody coupled to PLA-M magnetic microbeads that rely on immobilization are simple and more easily evaluated. Anti-HA tag antibody peroxidase labels have been used to enhance the sensitivity of immunoassays [26]. In the present study, a concentration of Y-scFv antibody of 2–4 mg/mL was coupled to the surface of the magnetic microbeads. The concentration of Y-scFv antibody had a highly significant effect on the efficiency of immobilization.

This novel method of sexing bovine sperm with MACS by using Y-scFv antibody can effectively separate X- and Y-sperm. This study showed the optimal level of Y-scFv coupling to the surface of PLA-M magnetic microbeads to be 2, 3, or 4 mg/mL, which demonstrated high-efficiency binding with Y-sperm and high accuracy of Y-sperm in the eluted fraction, with up to 78.01%, 78.63%, and 81.43%, respectively. Furthermore, this approach yielded a high proportion of X-sperm in the supernatant fraction, which represented the X-enriched fraction, up to 82.65%. These results showed that PY-microbeads bound to Y-sperm, and left X-sperm in the supernatant fraction, making the supernatant fraction an enriched X-sperm fraction with a low concentration of Y-sperm. Therefore, PY-microbeads bound to Y-sperm were trapped by the powerful neodymium magnet. Eluting buffer was used to separate the Y-sperm from the entrapped PY-microbeads. The Y-sperm were liberated from the PY-microbeads and remained in the eluted fraction. However, this fraction still had a low concentration of X-sperm because of the cross-reactivity of the Y-scFv antibody. The eluted fraction represented an enriched Y-sperm fraction. The success of this technology can be utilized to produce sex-selected sperm, allowing farmers to enhance the production of replacement female dairy cows.

Sperm motility is one of the most critical aspects of sperm fertility, as it is important for sperm transportation and fertilization in the female reproductive tract [27]. CASA provides a wide range of sperm motility parameters that provide valuable information regarding the physiological status of sperm and thus has the potential to more accurately predict sperm fertility than the parameters assessed by routine microscopic semen evaluation [28,29]. After the production of sexed semen by PY-microbeads, the NC- and X-enriched fractions showed normal motility, with kinematics that did not differ from those of CON. This means that PY-microbeads do not damage sperm. The present results were in agreement with those obtained by Domínguez et al. (2018) [30], who found that magnetic bead separation had no effect on donkey sperm motility. However, sperm in the Y-enriched fraction showed significantly decreased motility after sexing with PY-microbeads. The evaluation of acrosome integrity is critical because acrosome enzymes are important for penetrating the zona pellucida and fertilization. The acrosome integrity indicators of frozen–thawed sexed semen generated with PY-microbeads did not differ from those of CON in the current study. Moreover, the viability of sperm in the Y-enriched fraction was lower than in the X-enriched fraction. The elution buffer and duration of the procedure likely affect the Y–sperm in the Y-enriched fraction. Therefore, the low motility and low viability of the Y-enriched fraction need to be improved in future research.

In accordance with Thongkham et al. (2021) [7] imaging flow cytometry was used to discriminate X- and Y-chromosome-bearing sperm. Hoechst 33,342 is more concentrated in X-bearing sperm than in Y-bearing sperm, allowing them to be distinguished using imaging flow cytometry. Sperm containing X and Y chromosomes has a double Gaussian distribution on a histogram. In this study, the X-enriched fraction had a higher proportion of X-sperm. In this way, the use of X-enriched fraction semen would be almost certain to increase the percentage of female calves born by artificial insemination. Additionally, the proportion of sperm carrying the Y-chromosome increased in the Y-enriched fraction after sex selection. However, sperm viability for artificial insemination continues to be a challenge. Real-time PCR is regarded as a practical, accurate, and reliable method for quantifying spermatozoa carrying the X and Y chromosomes in bovine semen [31,32]. The confirmation of bovine sperm sexed by PY-microbeads was performed in this work by utilizing SYBR^®^ Green-based real-time quantitative PCR. During spermatogenesis, an equal number of sperm with both X and Y chromosomes are made, according to the meiotic model. The results of our real-time PCR validation of sexed bovine sperm are similar to previous investigations on bovine semen [33,34]. The 2 delta Ct value of X- and Y-chromosome-containing sperm differed significantly (3- to 4-fold) from the control. The SYBR^®^ Green real-time PCR technology applied in this work accurately determined the sperm sex ratio in terms of the fold increase for both X- and Y-enriched sperm.

## 5. Conclusions

This initial MACS, which used a Y-scFv antibody linked to the surface magnetic microbeads, was demonstrated to be an efficient approach for sexing bovine sperm. The optimal concentration of Y-scFv antibody coupled to magnetic microbeads was 2 mg/mL. The bound sperm on PY-microbeads was highly Y-sperm, and the unbound sperm was highly X-sperm. This approach generated sperm with up to 82.65% X-sperm in the X-enriched fraction and up to 81.43% Y-sperm in the Y-enriched fraction. Additionally, this method had no negative effect on X-sperm motility, kinetics, or quality; however, Y-sperm quality was low. PY-magnetic beads can be utilized to develop a technique for bovine X-sperm sexing.

## Figures and Tables

**Table 1:** Specific primers used to amplify bovine SRY, PLP and GAPDH.

Gene	Sequence (5′ → 3′)	Length (bp)	Accession No.
Y chromosome specific			
SRY-Forward	GAAAATAAGCACAAGAAAGTCCAGG	124	EU581861.1
SRY-Reverse	CAAAAGGAGCATCACAGCAGC		
X chromosome specific			
PLP-Forward	GGTGTGTTAGTTTCTGCTGTACAATAAATGG	96	AJ009913.1
PLP-Reverse	GATGGCAGGTGAGGGTAGGA		
Housekeeping gene			
GAPDH-Forward	GGCGCCAAGAGGGTCAT	120	NM_001034034.2
GAPDH—Reverse	GGTGGTGCAGGAGGCATT		

SRY: sex-determining region Y; PLP: proteolipid protein; GAPDH: glyceraldehyde-3-phosphate dehydrogenase.

**Table 2:** Percentage of X- and Y-sperm expressed in each fraction produced by different concentrations of Y-scFv antibody coupled to PLA-M microbeads (mean ± SD).

Concentration of Y-scFv Antibody (mg/mL)	Supernatant Fraction		Eluted Fraction	
	X-Sperm	Y-Sperm	X-Sperm	Y-Sperm
0	50.86 ± 0.58 ^a^	49.13 ± 0.58 ^d^	49.90 ± 0.60 ^b^	50.10 ± 0.60 ^a^
0.5	52.26 ± 1.15 ^ab^	47.73 ± 1.15 ^cd^	49.13 ± 0.66 ^b^	50.87 ± 0.66 ^a^
1	54.93 ± 0.64 ^b^	45.06 ± 0.64 ^c^	46.50 ± 0.60 ^b^	53.50 ± 0.60 ^a^
2	79.04 ± 0.15 ^c^	20.95 ± 0.15 ^b^	21.90 ± 0.95 ^a^	78.01 ± 0.95 ^b^
3	80.54 ± 0.61 ^cd^	19.46 ± 0.61 ^ab^	21.37 ± 3.06 ^a^	78.63 ± 3.06 ^b^
4	82.65 ± 0.87 ^d^	17.35 ± 0.87 ^a^	18.57 ± 1.00 ^a^	81.43 ± 1.00 ^b^

Different superscript letters in the same column indicate significant differences (*p* < 0.001).

**Table 3:** Evaluation of motility and kinematics of frozen–thawed sexed semen by PY-microbeads (*n* = 20; mean ± SD).

Parameter	Treatment				*p*-Value
	T1	T2	T3		
	CON	NC	(X-Enriched)	(Y-Enriched)	
TM (%)	68.79 ± 7.54 ^b^	64.90 ± 14.30 ^b^	62.90 ± 12.30 ^b^	23.55 ± 6.82 ^a^	0.030
PM (%)	57.11 ± 9.49 ^b^	54.12 ± 16.13 ^b^	52.14 ± 10.16 ^b^	15.17 ± 6.61 ^a^	0.021
VCL (µm/s)	89.52 ± 9.63 ^b^	79.22 ± 12.85 ^b^	74.36 ± 10.87 ^b^	26.04 ± 6.77 ^a^	0.040
VSL (µm/s)	35.69 ± 6.07 ^b^	33.55 ± 10.11 ^b^	31.25 ± 11.29 ^b^	11.97 ± 5.66 ^a^	0.001
VAP (µm/s)	45.25 ± 6.51 ^b^	42.01 ± 10.67 ^b^	40.24 ± 12.55 ^b^	14.89 ± 5.41 ^a^	0.001
DCL (µm)	30.03 ± 5.64 ^b^	27.25 ± 9.39 ^b^	24.11 ± 10.48 ^b^	12.25 ± 5.30 ^a^	0.010
DSL (µm)	10.53 ± 3.95 ^b^	9.09 ± 3.12 ^b^	8.02 ± 3.42 ^b^	5.00 ± 2.89 ^a^	0.024
DAP (µm)	12.37 ± 2.87 ^b^	11.34 ± 4.12 ^b^	10.42 ± 3.12 ^b^	6.47 ± 3.12 ^a^	0.041
ALH (µm)	0.90 ± 0.25 ^b^	0.81 ± 0.12 ^b^	0.79 ± 0.41 ^b^	0.43 ± 0.25 ^a^	0.025
BCF (Hz)	9.55 ± 5.45 ^b^	9.02 ± 0.44 ^b^	8.34 ± 3.69 ^b^	4.33 ± 2.27 ^a^	0.024
HAC (rad)	0.24 ± 0.15 ^b^	0.23 ± 0.11 ^b^	0.21 ± 0.07 ^b^	0.11 ± 0.08 ^a^	0.010
WOB (%)	0.52 ± 0.11 ^b^	0.53 ± 0.15 ^b^	0.51 ± 0.06 ^b^	0.56 ± 0.09 ^a^	0.008

Con: conventional frozen–thawed semen; NC: conventional semen incubated with non-active PLA-M microbeads; X-enriched: unbound sperm with PY-microbeads; Y-enriched: entrapped sperm with PY- microbeads. Different superscript letters in the same row indicate significant differences (*p* < 0.05).

**Table 4:** Acrosome integrity of frozen–thawed sexed semen by PY-microbeads (*n* = 20; mean ± SD).

Parameter	Treatment				*p*-Value
	T1	T2	T3		
	CON	NC	(X-Enriched)	(Y-Enriched)	
LI	42.40 ± 7.25 ^b^	40.20 ± 6.25 ^b^	38.90 ± 7.77 ^b^	12.44 ± 3.25 ^a^	0.001
DI	30.77 ± 4.42 ^a^	35.72 ± 5.52 ^a^	40.20 ± 7.25 ^a^	60.72 ± 9.01 ^b^	0.004
LR	0.77 ± 0.04 ^b^	0.82 ± 0.24 ^b^	0.44 ± 0.05 ^a^	0.42 ± 0.12 ^a^	0.032
DR	26.06 ± 2.94	23.26 ± 4.35	20.46 ± 8.88	26.42 ± 1.24	0.125

Con: conventional frozen–thaw semen; NC: conventional semen incubated with non-active PLA-M microbeads; X-enriched: unbound sperm with PY-microbeads; Y-enriched: entrapped sperm with PY- microbeads; LI: live-acrosome-intact sperm; LR: live acrosome-reacted sperm; DI: dead acrosome-intact sperm; DR: dead acrosome-reacted sperm. Different superscript letters in the same row indicate significant differences (*p* < 0.05).

## Data Availability

Not applicable.
